# Viral etiology and epidemiology of pediatric patients hospitalized for acute respiratory tract infections in Macao: a retrospective study from 2014 to 2017

**DOI:** 10.1186/s12879-021-05996-x

**Published:** 2021-03-26

**Authors:** Cheng Lei, Lisong Yang, Cheong Tat Lou, Fan Yang, Kin Ian SiTou, Hao Hu, King Io, Kun Tat Cheok, Baoquan Pan, Carolina Oi Lam Ung

**Affiliations:** 1 Department of Pediatrics, Kiang Wu Hospital, Macao SAR, China; 2 State Key Laboratory of Quality Research in Chinese Medicine, Institute of Chinese Medical Sciences, University of Macau, Macao SAR, China

**Keywords:** Viral etiology, Epidemiology, Respiratory viruses, Acute respiratory infections, Children, Macao

## Abstract

**Background:**

Acute respiratory infections (ARIs) are among the leading causes of hospitalization in children. Understanding the local dominant viral etiologies is important to inform infection control practices and clinical management. This study aimed to investigate the viral etiology and epidemiology of respiratory infections among pediatric inpatients in Macao.

**Methods:**

A retrospective study using electronic health records between 2014 and 2017 at Kiang Wu Hospital was performed. Nasopharyngeal swab specimens were obtained from hospitalized children aged 13 years or younger with respiratory tract diseases. xMAP multiplex assays were employed to detect respiratory agents including 10 respiratory viruses. Data were analyzed to describe the frequency and seasonality.

**Results:**

Of the 4880 children enrolled in the study, 3767 (77.1%) were positive for at least one of the 13 viral pathogens tested, of which 2707 (55.5%) being male and 2635 (70.0%) under 2 years old. Among the positive results, there were 3091 (82.0%) single infections and 676 (18.0%) multiple infections. The predominant viruses included human rhinovirus/enterovirus (HRV/EV 27.4%), adenovirus (ADV, 15.8%), respiratory syncytial virus B (RSVB, 7.8%) and respiratory syncytial virus A (RSVA, 7.8%). The detection of viral infection was the most prevalent in autumn (960/1176, 81.6%), followed by spring (1095/1406, 77.9%), winter (768/992, 77.4%), and summer (944/1306, 72.3%), with HRV/EV and ADV being most commonly detected throughout the 4 years of study period. The detection rate of viral infection was highest among ARI patients presented with croup (123/141, 87.2%), followed by lower respiratory tract infection (1924/2356, 81.7%) and upper respiratory tract infection (1720/2383, 72.2%). FluA, FluB and ADV were positive factors for upper respiratory tract infections. On the other hand, infection with RSVA, RSVB, PIV3, PIV4, HMPV, and EV/RHV were positively associated with lower respiratory tract infections; and PIV1, PIV2, and PIV3 were positively associated with croup.

**Conclusions:**

This is the first study in Macao to determine the viral etiology and epidemiology of pediatric patients hospitalized for ARIs. The study findings can contribute to the awareness of pathogen, appropriate preventative measure, accurate diagnosis, and proper clinical management of respiratory viral infections among children in Macao.

## Background

Acute respiratory infections (ARIs) are the leading infectious disease among children across the countries [1]. Being one of the most common causes to the overall health burden [2–5], ARIs are also significantly associated with hospitalization and death among children in China [6]. Children due to their weak physique and low immunity are susceptible to the clinical manifestations of fast transmissible and highly contagious viral ARIs [7]. Prolonged and serious infections in the immunocompromised host resulting in admission to the intensive care unit and death have been reported [8]. Reinfections associated with potentially severe illness in the children may occur throughout their life [9].

Despite the high burden of disease due to viral ARIs among children, it has been challenging to develop the best preventive or treatment measures [10–12]. Most of the evidence-based management guidelines suggest that there is no effective preventative or curative treatment for viral ARIs [13–15]. To inform and improve the successful implementation of prevention, control and treatment strategies, and the standardization of the diagnosis and clinical management of viral ARIs in children, it is important to have a good understanding of the etiological attributes and epidemiological characteristics among pediatric patients.

It has been suggested that the viral etiology of ARIs among pediatric patients vary across the regions due to the climate, geographical environment and population even within the same country [16–18]. For instance, in China, data showed that the most commonly detected virus causing ARIs in children was HRV in Chengdu (23%), RSV in Beijing (52.9%), HRV/EV in Shanghai (33.6%) [19–21]. Similar disparities have also been observed within the smaller region of the Pearl River Delta region. The detection rate was also different among neighboring areas due to the multifactorial etiology and diverse pathogens (Shenzhen: 14.55%, Guangzhou: 55.7%, Hong Kong: 64%) [22–24]. Located along the southeast coast of Mainland China, Macao (22°11′N, 113°33′E) is bordered on Guangdong Province and in close proximity of Hong Kong.

At present, little has been reported about the viral etiologies and epidemiology of pediatric patients suffering from ARIs in Macao. In this study, we retrospectively analyzed the pediatric patients hospitalized for ARIs from 2014 to 2017. The aim was to investigate the epidemiological characteristics and clinical features of the common respiratory viruses that cause ARIs, and to establish the respiratory viral pathogen spectrum among children to inform infection control practices and clinical management.

## Methods

A retrospective study was performed using data from the electronic health records of Kiang Wu Hospital about pediatric patients hospitalized for ARIs between 1 January 2014 and 31 December 2017*.*

### Study site

Macao has a typical subtropical Marine monsoon climate, characterized by rich heat, adequate moisture, high temperature, and abundant rainfall. In spring and summer, it is hot and rainy, while relatively less rainfall in autumn and winter. The 30-year statistics in Macao shows that the average annual temperature is 22.6 °C, the annual mean relative humidity is 78.8% and the total precipitation is 2058.1 mm [25].

### Data sources

The health records of patients were all from Kiang Wu Hospital which was a major hospital in Macao. Kiang Wu Hospital housed 755 or 47% (755/1604) of hospital beds in Macao, with its outpatient departments treated over 1.32 million patients, an average of 4028 per day to a population of 622,585 in 2017 [26, 27].

### Study population and data collection

The study population of the study was children aged from newborn to 13 years old who were hospitalized for diagnosed ARIs in Kiang Wu Hospital from 1st January 2014 and 31st December 2017. The files of all pediatric patients hospitalized admitted during the above mentioned period was extracted from the electron health records of Kiang Wu Hospital and screened by one of the authors (CL) using the corresponding ICD-10 codes: J00 (Acute nasopharyngitis [common cold]), J02 (Acute pharyngitis), J04 (Acute laryngitis and tracheitis), J06 (Acute upper respiratory infections of multiple and unspecified sites), J18 (Pneumonia, unspecified organism), and J20 (Acute bronchitis). The extracted data about eligible patients was then verified by another author (CTL). Both authors (CL and LCT) were specialists at the Department of Pediatrics. Two inclusion criteria were applied. The first criterion was that the patients were hospitalized with main diagnoses of ARIs. The second criterion was that the patient profiles must have complete results of the nasopharyngeal swab tests. Children with congenital pneumonia (ICD-10 code: P23), nosocomial infections (ICD-10 code: Y95) or chronic tuberculosis (ICD-10 code: A15) were excluded.

### Specimens

All the samples (*n* = 4880) collected from pediatric inpatients hospitalized for ARIs between 1st January 2014 and 31st December 2017 were enrolled to the study. The demographic, epidemiological and clinical information were extracted from the electronic health records of the hospital. The samples were collected from the patients at admission using a special swab (ESWAB from Copan Italia SpA) by the nurses. The swab was then put into culture medium, covered, and transported to the laboratory immediately at a storing temperature of 20–25 °C for testing within 1 h of collection.

### Laboratory procedure

Detection of respiratory viruses was performed immediately after the nasopharyngeal secretion specimens were delivered to the laboratory. Luminex 200, an xMAP equipment, combined with xTAG Respiratory Viral Panel FAST v2 were employed for the detection of viruses in this study. The device was selected due to its higher throughput, higher flexibility and better sensitivity and reproducibility when compared with traditional virus detection methods [28, 29]. It can detect multiple viral pathogens at the same time and even new viruses, a function with high clinical value considering the trend of multiple infection and gradual mutation of new virus.

All specimens were tested for 13 common respiratory viruses, including human rhino/enteroviruses (HRV/EV), adenovirus (ADV), respiratory syncytial virus A (RSVA), respiratory syncytial virus B (RSVB), human metapneumovirus (HMPV), influenza virus A (FluA), influenza virus B (FluB), human bocavirus (HBoV), human coronaviruses (HCoV), human parainfluenza virus 1 (PIV1), human parainfluenza virus 2 (PIV2), human parainfluenza virus 3 (PIV3), and human parainfluenza virus 4 (PIV4).

For nucleic acid extraction, after pretreatment (addition of 3.6ul cRNA and 56ul elution buffer AVE derived from Quagen GmbH), the samples were taken for nucleic acid extraction (including DNA and RNA) using an automated nucleic acid extractor, and the extracted nucleic acids were stored at − 70 °C. This was then followed by nucleic acid amplification during which the nucleic acid extraction solution was added to Master Mix (including RT-PCR Enzyme mix, Rnase free water, RT PCR buffer, Primer mix and dNTP mix) and placed in nucleic acid amplification apparatus for 15 pre-cycles to increase viral load, and then run 36 cycles. The viral nucleic acid amplification product was then placed in Luminex to detect whether there is a positive viral nucleic acid according to the manufacturer’s instructions.

### Variables

For this study, patient characteristic information included gender, age, season of admission and clinical diagnosis. Diagnosis of upper respiratory tract infections, croup and lower respiratory tract infections were made and confirmed by attending doctors at the Department of Pediatrics based on the patients’ clinical presentation. The viral pathogens were as reported in the results of the nasopharyngeal swab tests using xMAP multiplex assays.

### Data analysis

Excel and SPSS 24.0 statistical software were used for data processing and analysis, Chi-square test and Fisher’s test were used to compare the differences in classification variable distribution. Logistic regression analysis was used to analyze the relationship between independent predictors (viruses detected by the xTAG Respiratory Viral Panel FAST v2) and the outcome (clinical diagnosis made by the attending doctors) when *p* < 0.05 was considered statistically significant. To assess the goodness of fit of the logistic regression model, the Hosmer-Lemeshow test was used (*p* = 0.77 > 0.05, indicating no significant difference between the predicted value and the observed value), results of which suggested that the goodness of fit of the model was fair.

### Ethics statement

Ethics approval has been granted (No.2020–003) by the Research Ethics Committee of Kiang Wu Hospital. All the data were collected retrospectively and anonymized in a standardized case report form in the hospital database.

## Results

### Demographic characteristics and clinical diagnosis

In this study, 4880 pediatric patients hospitalized for ARIs admitted between January 1, 2014 and December 31, 2017 were recruited (Table 1). Among them, 3767 were tested positive for at least one virus for the respiratory viruses tested (77.2%). The prevalence of viral infection was higher among male (*n* = 2120, 56.3%) or patients aged between 1 to younger than 3 years old (*n* = 1962, 52.1%). Major clinical diagnosis of viral ARIs included lower respiratory tract infection (*n* = 1924, 51.1%) and upper respiratory tract infection (*n* = 1720, 45.7%). Overall, the admission rate of ARI patients test positive for viral infection peaked in spring during the study period (*n* = 1059, 29.1%).

**Table 1:** Demographic and clinical characteristics of study participants

Parameter	Patients with ARIs (*n* = 4880)		Viral infection			
			Yes (*n* = 3767)		No (=1113)	
	Counts	%	Counts	%	Counts	%
**Gender**						
Male	2707	55.5%	2120	56.3%	587	52.7%
Female	2173	44.5%	1647	43.7%	526	47.3%
**Age group (years)**						
0- < 1 (infant)	857	17.6%	673	17.9%	184	16.5%
1- < 3 (toddler)	2407	49.3%	1962	52.1%	445	40.0%
3- < 6 (preschool child)	1222	25.0%	908	24.1%	314	28.2%
≥ 6 (school child)	394	8.1%	224	5.9%	170	15.3%
**Season of admission**						
Spring	1406	28.8%	1095	29.1%	311	27.9%
Summer	1306	26.8%	944	25.1%	362	32.5%
Autumn	1176	24.1%	960	25.5%	216	19.4%
Winter	992	20.3%	768	20.4%	224	20.1%
**Clinical diagnosis**						
Upper respiratory tract infection	2383	48.8%	1720	45.7%	663	59.6%
Croup	141	2.9%	123	3.3%	18	1.6%
Lower respiratory tract infection	2356	48.3%	1924	51.1%	432	38.8%

### Viral etiology

The results about the viral etiology and the epidemiological data based on the patients in this study are reported in the following in terms of the viral distribution by gender, age, hospital admission time and clinical diagnosis (with more detailed information provided in the Supplement).

### Overall viral etiology

The most common viruses detected were HRV/EV (*n* = 1336, 35.5%), ADV (*n* = 771, 20.5%), RSVB (*n* = 379, 10.1%), RSVA (*n* = 376, 10.0%), HMPV (*n* = 334, 8.9%) and Flu A (*n* = 302, 8.0%) (Table 2). Among the viral infection cases, 3091 cases were single infection (82.1%) and 676 cases were multiple viruses infection cases (17.9%) which were mostly double-virus infection (*n* = 656, 97.0%). The viruses most commonly involved in multiple-virus infection included HRV/EV (*n* = 457), ADV (*n* = 226), and HBoV (*n* = 159).

**Table 2:** Infection situation of 13 common respiratory viruses from Jan 2014 to Dec 2017 in Macao

Total (*N* = 4880)	Positive	HRV/EV	ADV	RSVB	RSVA	HMPV	FluA	HBoV	PIV3	FluB	PIV1	HCoV	PIV4	PIV2
**Total infection cases (n)**	3767	1336	771	379	378	334	302	251	237	139	134	131	44	29
**Total infection cases (%)**	77.2%	27.4%	15.8%	7.8%	7.8%	6.8%	6.2%	5.1%	4.9%	2.9%	2.8%	2.7%	0.9%	0.6%
Single infection cases (n)	3091	866	536	294	292	265	256	82	154	130	98	66	28	24
Single infection cases (%)	82.0%	64.8%	69.5%	77.6%	77.3%	79.3%	84.8%	32.7%	65.0%	93.5%	73.1%	50.4%	63.6%	82.8%
Multiple infection cases (n)	676	470	235	85	86	69	46	169	83	9	36	65	16	5
Multiple infection cases (%)	18.0%	35.2%	30.8%	22.4%	22.8%	18.3%	15.2%	67.3%	35.0%	6.5%	26.9%	49.6%	36.4%	17.2%
Multiple infection with 2 viruses (n)	656	457	226	80	82	66	44	159	77	9	36	59	13	4
Multiple infection with 3 viruses (n)	18	11	8	5	4	2	2	8	5	0	0	5	3	1
Multiple infection with 4 viruses (n)	2	2	1	0	0	1	0	2	1	0	0	1	0	0

#### Viral etiology and gender

**Fig. 1:**
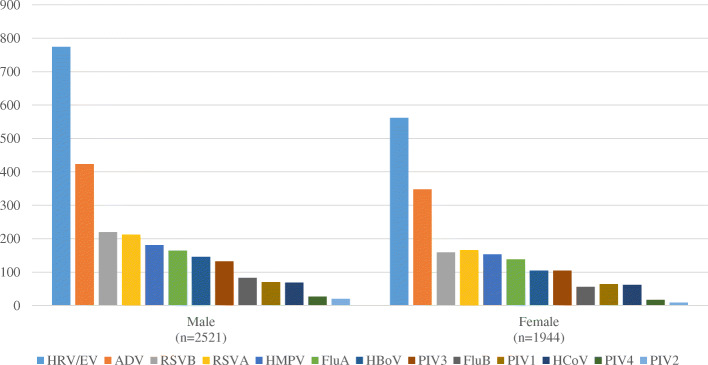
Viruses distribution among hospitalized ARI patients tested positive for viral infection (gender)

The detection rate of viral infection appear to be comparable between male (2120/2707, 78.3%) and female patients (1647/2173, 75.8%) (Fig. 1). The most common viruses among patients with viral infection were: *for male*, HRV/EV (*n* = 774/2521, 30.7%), ADV (*n* = 423/2521, 16.8%), RSVB (*n* = 220/2521, 8.7%) and RSVA (*n* = 212/2621, 8.4%), *and for female*, HRV/EV (*n* = 562/1944, 28.9%), ADV (*n* = 348/1944, 17.9%), RSVA (*n* = 166/1944, 8.5%) and RSVB (*n* = 159/1944, 8.2%).

#### Viral etiology and age

**Fig. 2:**
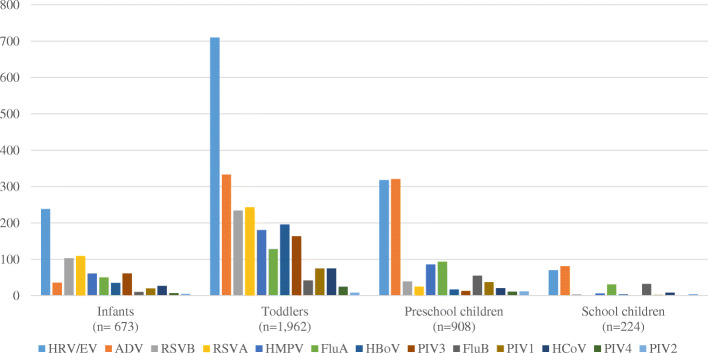
Viruses distribution among hospitalized ARI patients tested positive for viral infection (age groups)

The detection rate of viral infection was highest among toddlers (1962/2407, 81.5%), followed by infants (673/857, 78.5%), preschool children (908/1222, 74.3%) and school children (224/394, 56.9%) (Fig. 2). The most common viruses in each age group were: *for infants*, HRV/EV (*n* = 238/673, 35.3%), RSVA (*n* = 109/673, 16.2%) and RSVB (*n* = 103/673, 15.3%); *for toddlers*, HRV/EV (*n* = 710/1962, 36.2%), ADV (*n* = 333/1962, 17.0%) and RSVA (*n* = 243/1962, 12.4%); *for preschool children*, ADV (*n* = 321/908, 35.4%), HRV/EV (*n* = 318/908, 35.0%) and FluA (*n* = 93/908, 10.2%); and *for school children*, ADV (*n* = 81/224, 36.2%), HRV/EV (*n* = 70/224, 31.3%) and FluB (*n* = 32/224, 14.3%). HRV/EV was the most common virus detected across all age groups, and so was ADV (except for infants).

#### Viral etiology and seasonality

**Fig. 3:**
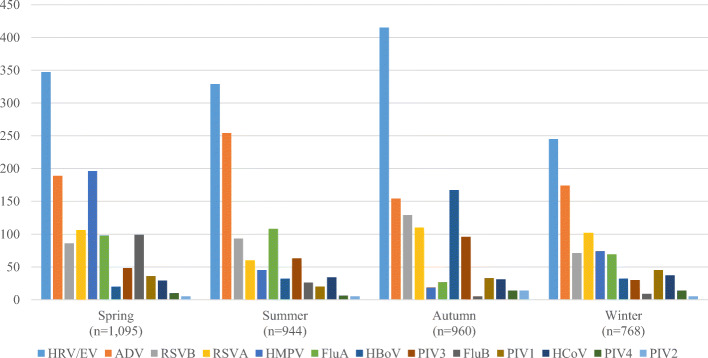
Viruses distribution among hospitalized ARI patients tested positive for viral infection (season)

The detection rate of viral infection was the highest in autumn (960/1176, 81.6%), followed by spring (1095/1406, 77.9%), winter (768/992, 77.4%), and summer (944/1306, 72.3%) (Fig. 3). The most common viruses detected in each season included: *for spring*, HRV/EV (347/1095, 31.7%), HMPV (*n* = 196/1095, 17.9%), and ADV (*n* = 189/1095, 17.3%); *for summer*, HRV/EV (*n* = 329/944, 34.9%), ADV (*n* = 254/944, 26.9%) and Flu A (*n* = 108/944, 11.4%); *for autumn*, HRV/EV (*n* = 415/960, 43.2%), HBoV (*n* = 167/960, 17.4%) and ADV (*n* = 154/960, 16.0%); and *for winter*, HRV/EV (*n* = 245/768, 31.9%), ADV (*n* = 174/768, 22.7%), and RSVA (*n* = 102/768, 13.3%) (Fig. 3). HRV/EV and ADV were the most common viruses detected across all 4 seasons.

**Fig. 4:**
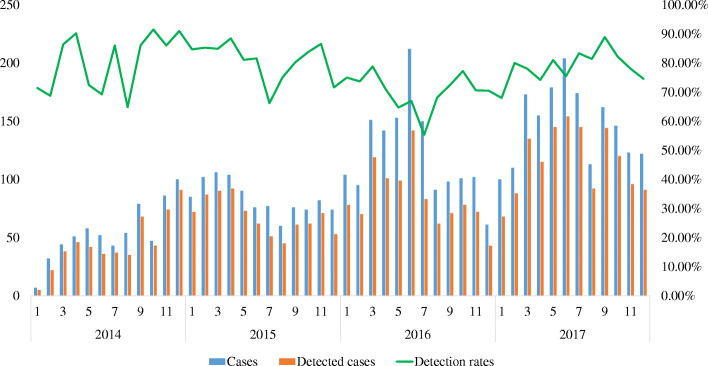
Monthly distribution of 13 common respiratory viruses in Macao

The monthly distribution of virus detection rate over the past 4 years showed that viruses could be detected in all months of the study period and demonstrated a seasonality pattern. In general, the number of hospitalized children for ARIs was the highest in spring (*n* = 1406) and summer (*n* = 1306), but the virus detection rate was the highest in autumn (960/1176, 81.6%) and the lowest in summer (944/1306, 72.3%) (X2 = 31.465, *p* < 0.001) (Fig. 4).

#### Viral etiology and clinical diagnosis

**Fig. 5:**
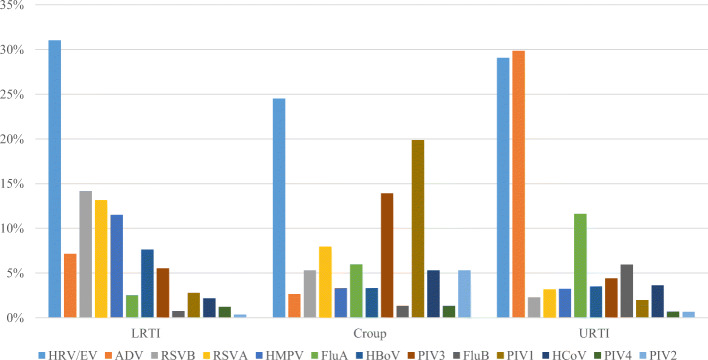
Detection rate of viruses for the 3 diagnosis among hospitalized ARI patients (percentage)

Overall, The detection rate of viral infection was highest among ARI patients presented with croup (123/141, 87.2%), followed by lower respiratory tract infections (LRTIs) (1924/2356, 81.7%) and upper respiratory tract infections (URTIs) (1720/2383, 72.2%). The most common viruses detected in each of the clinical diagnosis were: for croup, HRV/EV (25%), PIV1 (20%) and PIV3 (14%); for lower respiratory tract infection, HRV/EV (31%), RSVB (14%), RSVA (13%) and HMPV (12%); and for upper respiratory tract infection, ADV (30%), HRV/EV (29%) and Flu A (12%) (Fig. 5).

Logistic regression analysis showed that infection with FluA, FluB and ADV were positive factors for URTIs. On the other hand, infection with RSVA, RSVB, PIV3, PIV4, HMPV, and EV/RHV were positively associated with LRTIs; and PIV1, PIV2, and PIV3 were positively associated with croup as shown in Table 3.

**Table 3:** Logistic regression analysis of associations between respiratory viruses and clinical diagnosis

Virus	Upper respiratory tract infections (*N* = 2383)		Croup (*N* = 141)		Lower respiratory tract infections (*N* = 2356)	
	n (%)	OR (95% CI)	n (%)	OR (95% CI)	n (%)	OR (95% CI)
RSVA	64 (1.3%)	0.16 (0.12–0.21)			302 (6.2%)	5.50 (4.20–7.20)
RSVB	46 (0.9%)	0.11 (0.08–0.15)			325 (6.7%)	8.40 (6.19–11.40)
FluA	235 (4.8%)	2.88 (2.15–3.86)			58 (1.2%)	0.31 (0.23–0.42)
FluB	120 (2.5%)	4.89 (2.98–8.04)			17 (0.3%)	0.19 (0.11–0.32)
PIV1	40 (0.8%)	0.31 (0.21–0.46)	30 (0.6%)	17.20 (10.30–28.75)		
PIV2			8 (0.2%)	22.22 (9.24–53.47)		
PIV3	89 (1.8%)	0.52 (0.39–0.69)	21 (0.4%)	5.34 (3.13–9.12)	127 (2.6%)	1.40 (1.06–1.85)
PIV4	14 (0.3%)	0.37 (0.19–0.72)			28 (0.8%)	2.38 (1.26–4.50)
HMPV	65 (1.3%)	0.18 (0.14–0.24)			264 (5.4%)	5.43 (4.09–7.20)
HRV/EV	587 (12.0%)	0.63 (0.55–0.73)			712 (14.6%)	1.54 (1.33–1.79)
ADV	603 (12.4%)	3.41 (2.81–4.14)	4 (0.1%)	0.22 (0.08–0.61)	164 (3.4%)	0.31 (0.26–0.38)
HCoV			8 (0.2%)	3.60 (1.67–7.73)	50 (1.0%)	0.63 (0.42–0.92)
HBoV	71 (1.5%)	0.37 (0.28–0.50)			175 (3.6%)	2.70 (2.01–3.61)

Only the variables with *p* < 0.05 were shown

## Discussion

This study investigated the viral etiology and epidemiology of pediatric patients hospitalized for ARIs in Macao for 4 consecutive years and confirmed the importance of respiratory viruses among the children population. Of the 4880 patients admitted in this study, 77.2% tested positive for at least 1 virus, and the principal pathogens were HRV/EV (35.5%), ADV (20.5%), RSV B (10.1%) and RSV A (10.0%). These findings are different from what had been reported in areas nearby [22–24] affirming the justification for this study. At least 4 out of 5 toddlers (1- < 3 years old) tested positive for viral infection representing more than half of viral infections (52.1%) which was in line with previous studies [22, 23, 30]. It is also worth noting that in this study: (1) HRV/EV was the common pathogen and caused most of the upper or lower respiratory tract infections; (2) RSVA and RSVB mostly affected patients under 3 years old and were more likely to cause lower respiratory tract infections; and (3) ADV were mostly found in patients aged 1 year old or older and was more likely to cause upper respiratory tract infections.

The detection of respiratory viruses among hospitalized pediatric patients in Macao reached 77.1% (3767/4880), which is much higher than that was reported in neighboring areas such as Guangzhou (55.7% or 2361/4242) [22] and Shenzhen (14.55% or 4428/30,443) [23], and other parts of China such as Shanghai (24.5% or 691/2819) [30] and Chengdu (51% or 900/1764) [21]. Although different study designs (such as patient age groups, detect method, virus typing and admission criteria, etc.) prevented direct comparisons of the findings, the small geographical territory, high population density, immune status, age structure, virus contact rates and climate characteristics of abundant heat and water vapor may help explain the discrepancies [31].

Considering that respiratory viruses are spread via contact, droplet, and aerosol transmission [32], modifiable factors such as proper personal and environmental hygiene practice are crucial prompting the need for public education. Moreover, knowledge about respiratory viral etiology is also important to the differentiation between bacterial infections and viral infections [33]. The use of antibiotics in viral infections does not improve the clinical outcomes but will instead deteriorates the issues around antimicrobial resistance. Decreasing the use of unnecessary antibiotics is the key to combating antimicrobial resistance [34]. Informing clinicians’ decision about rational prescribing of antibiotics with the local viral etiology characteristics not only benefits patients’ clinical outcomes but will also contribute to a reduction of resistance [35]. Moreover, the high detection rate of virus may pose a serious threat to the public health warranting an effective and rigorous surveillance system to allow early detection and timely management [36].

Amongst children ill enough from ARIs to be hospitalized in this study, HRV/EV were the most frequently identified viruses. This was supported by previous studies that revealed HRV/EV was the common pathogens responsible for respiratory infections among children [37, 38]. Although HRV/EV was traditionally associated with upper respiratory tract disease, this study shows that HRV/EV was also associated with lower respiratory tract disease [39]. These findings can be used to support clinicians’ decisions about careful and timely management of the respiratory conditions in order to prevent substantial morbidity associated with severe viral respiratory infection due to HRV/EV in children as reported in previous studies [40].

The findings that RSVB and RSVA were also major etiology in young hospitalized patients with ARIs were consistent with study results from China and other countries [41, 42]. RSV with lower respiratory tract infections are a major worldwide threat with a high socioeconomic burden for children under 5 years of age [13]. This indicated that the prevention strategies for RSV infection could have an important implications for public health in Macao. At present, palivizumab, a monoclonal antibody used in the prevention of RSV infection, is available. Evidence has suggested that palivizumab as RSV prophylaxis is cost-effective [43] but is nevertheless expensive and may need to be paid out-of-pocket. Current evidence-based global management guidelines also showed that challenges remain for their clinical prevention and treatment [15, 44, 45]. Therefore, more support is needed to enable children especially those at high risk of viral infection to receive the treatment to prevent serious upper and lower respiratory tract infections.

This study also shows that ADV respiratory infections were common especially for those aged between 1 and 5 years, which is similar to previous study reports [46, 47]. The present data also showed that ADV infections affected boys and girls similarly while previous studies have suggested otherwise [48]. For this, more extensive viral etiology studies would be needed to investigate if gender is indeed a factor with regards to ADV infections. On the other hand, Flu are worth further discussion as they are a major cause of morbidity and mortality for children especially those with certain comorbidities [49, 50]. The detection rate of Flu A/B among hospitalized children with ARIs was estimated at 9.1% in this study, which appeared to be higher than 8.9% in Beijing and Shanghai [20] and the global estimates reported in a systematic review of 100 studies which found that influenza virus accounted for 5% of acute lower respiratory infection hospital admissions [51]. Although direct comparison of these results is not appropriate due to disparities in the study design and variations in influenza activity across different times, regions and populations, it is safe to say that Flu remains one of the important health threats to pediatric population in Macao. In order to reduce influenza-related infections and hospitalization, the Macao SAR Government promotes seasonal flu vaccination and provides free influenza vaccines to people aged from 6 months to 18 years, 50 years or above and other high-risks populations [52]. The Health Bureau even sends medical teams to schools and nurseries to deliver flu vaccine to both students and teachers. Nevertheless, the vaccination rate for children is not 100% and the number of children infected with influenza viruses requiring hospitalization remained relevant. For this, both hospital-based [53] and community-based interventions should be reinforced to promote the uptake of influenza vaccines among children to prevent influenza infections and related hospitalization [54].

In terms of multiple infections, 17.95% of the pediatric patients in this study were infected with at least two viruses involving mostly HRV/EV, ADV and HBoV. Similarly, multiple infection cases have also been reported elsewhere such as Guangzhou (21.3%) [22], and Shantou (46.9%) [55]. While some studies suggested that multiple infections were associated with increased hospital admissions, intensive care unit admissions, lengthened duration of hospitalization, and prolonged mechanical ventilation use [56–58], some other studies concluded that there was no significant difference between children with single and multiple respiratory viral infections in terms of the clinical disease severity, the length of hospital stay, admission to the intensive care unit, need for mechanical ventilation, oxygen requirements, and death [59–61].

The seasonality was also another attribute about the viral etiology observed in this study. Overall, the virus detection peaked in spring and was at trough in autumn. Detailed analysis of single virus showed that the prevalence of HRV/EV was persistently high throughout the whole year, with ADV peaked in summer and winter, and RSV peaked in autumn and winter. Similar findings have also been reported about China [23, 62] and Belgium [63]. However, the detection rate of influenza viruses was mainly prevalent in spring and summer, which were very different from the research findings even in neighboring areas [22, 24]. This indicated that even in the same climate zone, different areas might have different virus epidemic characteristics. Knowledge about influenza viruses seasonality dynamics is exceptionally important for informing the optimal schedule for vaccination [64, 65] and the rational inventory and use of antivirals [66–68]. For the influenza vaccine or other new virus vaccines, low incidence seasons may represent a target window for vaccination so that the population immunity may be maximized before the onset of the influenza season, and the number of severe outcomes associated with viral infection may be minimized [69]. Whenever the prophylactic treatment is available, the first dose should be given before the season starts [70].

There were some limitations in this study. Firstly, HRV and EV belong to the same virus family, most common detection equipment does not separate the two viruses [24, 62]. The equipment of our study also put them in the same category, so they can only be studied by combining data. Secondly, no outpatient cases were included in this study, which would affect the integrity of the pathogen profile. Kiang Wu hospital patient population might only represent half of the Macao patient population. The findings of this study can serve a good indication about population-based viral etiology among children in Macao and it can provide important reference for local further research or Pearl River Delta which has a similar geographical environment and population structure. In the further research, we will continue to investigate the association of virus epidemic with meteorological factors and air pollutants, to seek reasonable explanations. Due to the nature of the dataset available for this study, only retrospective and descriptive analysis was possible which fell short to test any hypotheses to address some of the utmost knowledge gaps regarding pathogens. However, having this first study about viral etiology among pediatric inpatients in Macao, future studies have been planned to investigate the association between viral-viral co-infection and severity of ARIs, and the impact of influenza vaccination on the incidence of ARIs.

## Conclusions

In conclusion, to the best of our knowledge, our study is the first study in Macao to determine the viral etiology and epidemiology of pediatric patients hospitalized for ARIs. The study findings can contribute to the awareness of pathogen, appropriate preventative measure, accurate diagnosis, and proper clinical management of respiratory viral infections among children in Macao. At the same time, the information may contribute to the authorities for setting up future public health plans and guide further research in this area.

## Data Availability

The datasets used and/or analyzed during the current study are available from the corresponding author on reasonable request.

## Abbreviations

ARIs: Acute respiratory infections
HRV/EV: Human rhino/enteroviruses
ADV: Adenovirus
RSVA: Respiratory syncytial virus A
RSVB: Respiratory syncytial virus B
HMPV: Human metapneumovirus
FluA: Influenza virus A
FluB: Influenza virus B
HBoV: Human bocavirus
HCoV: Human coronaviruses
PIV1: Human parainfluenza virus 1
PIV2: Human parainfluenza virus 2
PIV3: Human parainfluenza virus 3
PIV4: Human parainfluenza virus 4

## References

[1] Forum of International Respiratory Societies (FIRS) (2014). Respiratory diseases in the world: realities of today—opportunities for tomorrow.

[2] Bryce J, Boschi-Pinto C, Shibuya K, Black RE (2005). WHO child health epidemiology reference group. WHO estimates of the causes of death in children. Lancet.

[3] Vos T, Barber RM, Bell B (2015). Global, regional, and national incidence, prevalence, and years lived with disability for 301 acute and chronic diseases and injuries in 188 countries, 1990–2013: a systematic analysis for the Global Burden of Disease Study 2013. Lancet.

[4] Abubakar II, Tillmann T, Banerjee A (2015). Global, regional, and national age-sex specific all-cause and cause-specific mortality for 240 causes of death, 1990-2013: a systematic analysis for the global burden of disease study 2013. Lancet.

[5] Ferkol T, Schraufnagel D (2014). The global burden of respiratory disease. Ann Am Thorac Soc.

[6] Rudan I, Chan KY, Zhang JS (2010). Causes of deaths in children younger than 5 years in China in 2008. Lancet.

[7] Manoha C, Espinosa S, Aho SL, Huet F, Pothier P (2007). Epidemiological and clinical features of hMPV, RSV and RVs infections in young children. J Clin Virol.

[8] Chatzis O, Darbre S, Pasquier J, Meylan P, Manuel O, Aubert JD, et al. Burden of severe RSV disease among immunocompromised children and adults: a 10 year retrospective study. BMC Infect Dis. 2018;18(1):111. 10.1186/s12879-018-3002-3. 10.1186/s12879-018-3002-3 PMC5838875 29510663

[9] Anderson EJ, Simões EA, Buttery JP (2012). Prevalence and characteristics of human metapneumovirus infection among hospitalized children at high risk for severe lower respiratory tract infection. J Pediatr Infect Dis Soc.

[10] Campbell H, Bont L, Nair H. Respiratory syncytial virus (RSV) disease - new data needed to guide future policy. J Glob Health. 2015;5(2):020101(1–4). 10.7189/jogh.05.020101 PMC4693507 26755941

[11] Afkhami S, Yao Y, Xing Z (2016). Methods and clinical development of adenovirus-vectored vaccines against mucosal pathogens. Mol Ther Methods Clin Dev.

[12] Ren J, Phan T, Bao X (2015). Recent vaccine development for human metapneumovirus. J Gen Virol.

[13] Mazur NI, Martinón-Torres F, Baraldi E (2015). Lower respiratory tract infection caused by respiratory syncytial virus: current management and new therapeutics. Lancet Respir Med.

[14] Meissner HC (2016). Viral bronchiolitis in children. N Engl J Med.

[15] Ralston SL, Lieberthal AS, Meissner HC (2014). Clinical practice guideline: the diagnosis, management, and prevention of bronchiolitis. Pediatrics.

[16] Van den Hoogen BG, de Jong JC, Groen J (2001). A newly discovered human pneumovirus isolated from young children with respiratory tract disease. Nat Med.

[17] Williams JV, Edwards KM, Weinverg GA (2010). Population-based incidence of human metapneumovirus infection among hospitalized children. J Infect Dis.

[18] Allander T, Tammi MT, Eriksson M, Bjerkner A, Tiveljung-Lindell A, Andersson B (2005). Cloning of a human parvovirus by molecular screening of respiratory tract samples. Proc Natl Acad Sci U S A.

[19] Yu J, Xie Z, Zhang T, Lu Y, Fan H, Yang D, et al. Comparison of the prevalence of respiratory viruses in patients with acute respiratory infections at different hospital settings in North China, 2012–2015. BMC Infect Dis. 2018;18(1):72. 10.1186/s12879-018-2982-3. 10.1186/s12879-018-2982-3 PMC5806372 29422011

[20] Zhao Y, Lu R, Shen J (2019). Comparison of viral and epidemiological profiles of hospitalized children with severe acute respiratory infection in Beijing and Shanghai, China. BMC Infect Dis.

[21] Chen J, Hu P, Zhou T, Zheng T, Zhou L, Jiang C, et al. Epidemiology and clinical characteristics of acute respiratory tract infections among hospitalized infants and young children in Chengdu, West China, 2009–2014. BMC Pediatr. 2018;18(1):216. 10.1186/s12887-018-1203-y. 10.1186/s12887-018-1203-y PMC6034247 29976175

[22] Liu W, Liu Q, Chen D (2014). Epidemiology of acute respiratory infections in children in Guangzhou: a three-year study. PLoS One.

[23] Wang H, Zheng Y, Deng J, Wang W, Liu P, Yang F, et al. Prevalence of respiratory viruses among children hospitalized from respiratory infections in Shenzhen, China. Virol J. 2016;13(1):39. 10.1186/s12985-016-0493-7. 10.1186/s12985-016-0493-7 PMC4782311 26952107

[24] Cowling BJ, Chan KH, Peiris JS (2018). Influenza-like illness and viral aetiology in Hong Kong children. Hong Kong Med J.

[25] Macao Meteorological and Geophysical Bureau. Climate in Macao. Available at: http://www.smg.gov.mo/smg/climate/e_climaintro.htm. Accessed 1 Sept 2019.

[26] Statistics and Census Service (2018). Health Statistics.

[27] Statistics and Census Service (2018). Health Statistics.

[28] Arellano-Garcia ME, Hu S, Wang J, Henson B, Zhou H, Chia D, et al. Multiplexed immunobead-based assay for detection of oral cancer protein biomarkers in saliva. Oral Dis. 2008;14(8):705–12. 10.1111/j.1601-0825.2008.01488.x. 10.1111/j.1601-0825.2008.01488.x PMC2675698 19193200

[29] Linkov F, Yurkovetsky Z, Lokshin A (2009). Hormones as biomarkers: practical guide to utilizing luminex technologies for biomarker research. Methods Mol Biol.

[30] Dong W, Chen Q, Hu Y, He D, Liu J, Yan H, et al. Epidemiological and clinical characteristics of respiratory viral infections in children in Shanghai, China. Arch Virol. 2016;161(7):1907–13. 10.1007/s00705-016-2866-z. 10.1007/s00705-016-2866-z PMC7086729 27138548

[31] Hu H, Nigmatulina K, Eckhoff P (2013). The scaling of contact rates with population density for the infectious disease models. Math Biosci.

[32] Kutter J, Spronken M, Fraaij P (2018). Transmission routes of respiratory viruses among humans. Curr Opin Virol.

[33] Lin C, Hwang D, Chiu N (2020). Increased detection of viruses in children with respiratory tract infection using PCR. Int J Environ Res Public Health.

[34] Shiley K, Lautenbach E, Lee I (2010). The use of antimicrobial agents after diagnosis of viral respiratory tract infections in hospitalized adults: antibiotics or anxiolytics?. Infect Control Hosp Epidemiol.

[35] Llor C, Bjerrum L (2014). Antimicrobial resistance: risk associated with antibiotic overuse and initiatives to reduce the problem. Ther Adv Drug Safety.

[36] Zhao H, Green H, Lackenby A (2014). A new laboratory-based surveillance system (respiratory DataMart system) for influenza and other respiratory viruses in England: results and experience from 2009 to 2012. Eurosurveillance.

[37] Messacar K, Robinson CC, Bagdure D (2013). Rhino/enteroviruses in hospitalized children: a comparison to influenza viruses. J Clin Virol.

[38] Spaeder M, Custer J, Miles A (2015). A multicenter outcomes analysis of children with severe rhino/enteroviral respiratory infection. Pediatr Crit Care Med.

[39] Messacar K, Robinson C, Bagdure D (2013). Rhino/enteroviruses in hospitalized children: a comparison to influenza viruses. J Clin Virol.

[40] Spaeder M, Custer J, Bembea M (2013). A multicenter outcomes analysis of children with severe viral respiratory infection due to human metapneumovirus. Pediatr Crit Care Med.

[41] Liu T, Li Z, Zhang S (2015). Viral etiology of acute respiratory tract infections in hospitalized children and adults in Shandong Province, China. Virol J.

[42] Stein RT, Bont LJ, Zar H, Polack FP, Park C, Claxton A, et al. Respiratory syncytial virus hospitalization and mortality: systematic review and meta-analysis. Pediatr Pulmonol. 2017;52(4):556–69. 10.1002/ppul.23570. 10.1002/ppul.23570 PMC5396299 27740723

[43] Mac S, Sumner A, Duchesne S (2019). Cost-effectiveness of Palivizumab for respiratory syncytial virus: A systematic review. Pediatrics.

[44] Turner T, Wilkinson F, Harris C (2008). Evidence based guideline for the management of bronchiolitis. Aust Fam Physician.

[45] National Institute for Health and Care Excellence (2015). Bronchiolitis in children: diagnosis and management.

[46] Tabain L, Ljubin-Sternak S (2012). Adenovirus respiratory infections in hospitalized children: clinical findings in relation to species and serotypes. Pediatr Infect Dis J.

[47] Yeung R, Eshaghi A, Lombos E, Blair J, Mazzulli T, Burton L, et al. Characterization of culture-positive adenovirus serotypes from respiratory specimens in Toronto, Ontario, Canada: September 2007-June 2008. Virol J. 2009;6(1):11. 10.1186/1743-422X-6-11. 10.1186/1743-422X-6-11 PMC2656483 19171030

[48] Dominguez O, Rojo P, de Las HS (2005). Clinical presentation and characteristics of pharyngeal adenovirus infections. Pediatr Infect Dis J.

[49] Cromer D, van Hoek AJ, Jit M (2014). The burden of influenza in England by age and clinical risk group: a statistical analysis to inform vaccine policy. J Inf Secur.

[50] Kalligeros M, Shehadeh F, Mylona E (2020). Influenza vaccine effectiveness against influenza-associated hospitalization in children: A systematic review and meta-analysis. Vaccine.

[51] Wang X, Li Y, O'Brien K, Madhi S (2020). Global burden of respiratory infections associated with seasonal influenza in children under 5 years in 2018: a systematic review and modelling study. Lancet Glob Health.

[52] Director of the Department of Social and Cultural Affairs. Dr. Alexis Tam Chong Weng, led the staff of the Health Bureau to inoculate the winter flu vaccine to build an immune barrier: Government Information Bureau; 2019. Assessed 5 Oct 2019. Available from: https://news.gov.mo/detail/zh-hans/N19JCHwqpM?1

[53] Ma V, Palasanthiran P, Seale H (2019). Exploring strategies to promote influenza vaccination of children with medical comorbidities: the perceptions and practices of hospital healthcare workers. BMC Health Serv Res.

[54] Alsaleh NA. Pharmacist-led flu vaccination services in community pharmacy: experiences and benefits. J Adv Pharm Educ Res. 2020;10(1):181–5.

[55] Cui B, Zhang D, Pan H, Zhang F, Farrar J, Law F, et al. Viral aetiology of acute respiratory infections among children and associated meteorological factors in southern China. BMC Infect Dis. 2015;15(1):124. 10.1186/s12879-015-0863-6. 10.1186/s12879-015-0863-6 PMC4365542 25884513

[56] Chauhan JC, Slamon NB (2017). The impact of multiple viral respira-tory infections on outcomes for critically Ill children. Pediatr Crit Care Med.

[57] Goka E, Vallely P, Mutton K (2014). Single and multiple respiratory virus infections and severity of respiratory disease: a systematic review. Paediatr Respir Rev.

[58] Yen C, Wu W, Chang C (2019). Viral etiologies of acute respiratory tract infections among hospitalized children–A comparison between single and multiple viral infections. J Microbiol Immunol Infect.

[59] Scotta M, Chakr V, Moura A (2016). Respiratory viral coinfection and diseaseseverity in children: a systematic review and meta-analysis. J Clin Virol.

[60] Asner S, Science M, Tran D (2014). Clinical disease severity of respiratory viral co-infection versussingle viral infection: a systematic review and meta-analysis. PLoS One.

[61] Lim J, Klerk N, Blyth C (2016). Systematic review and meta-analysis of respiratory viral coinfections in children. Respirology.

[62] Zhang C, Zhu N, Xie Z, Lu R, He B, Liu C, et al. Viral etiology and clinical profiles of children with severe acute respiratory infections in China. PLoS One. 2013;8(8):e72606. 10.1371/journal.pone.0072606. 10.1371/journal.pone.0072606 PMC3750056 23991128

[63] Cattoir L, Vankeerberghen A, Boel A, van Vaerenbergh K, de Beenhouwer H (2019). Epidemiology of RSV and hMPV in Belgium: a 10-year follow-up. Acta Clin Belg.

[64] Saha S, Chadha M, Al Mamun A, Sturm-Ramirez M, Chittaganpitch M (2014). Influenza seasonality and vaccination timing in tropical and subtropical areas of Southern and South-Eastern Asia. Bull World Health Organ.

[65] Lambach P, Alvarez A, Hirve S (2015). Considerations of strategies to provide influenza vaccine year round. Vaccine.

[66] Nguyen N Do, A. Chandna V, Nguyen C, et al. (2013). Antibiotic use and resistance in emerging economies: a situation analysis for Viet Nam. BMC Public Health.

[67] Laxminarayan R, Matsoso P, Pant S, Brower C, Røttingen JA, Klugman K, et al. Access to effective antimicrobials: a worldwide challenge. Lancet. 2016;387(10014):168–75. 10.1016/S0140-6736(15)00474-2. 10.1016/S0140-6736(15)00474-2 26603918

[68] Van T, Gandra S, Ashok A (2014). Global antibiotic consumption 2000 to 2010: an analysis of national pharmaceutical sales data. Lancet Infect Dis.

[69] Diseases C (2009). Modified recommendations for use of Palivizumab for prevention of respiratory syncytial virus infections. Pediatrics.

[70] European Medicines Agency (2013). European public assessment report (EPAR) for Synagis.

